# Delousing performance of ballan wrasse ( *Labrus bergylta* ) and lumpfish ( *Cyclopterus lumpus* ): seasonal consistency and the challenge of cryptic lice for lumpfish

**DOI:** 10.1002/ps.70582

**Published:** 2026-01-28

**Authors:** Adam Jonathan Brooker, Andrew Davie, David Bassett, Sally Boyd, Hervé Migaud

**Affiliations:** ^1^ Institute of Aquaculture University of Stirling Stirling UK

**Keywords:** aquaculture, sea lice, cleaner fish, pigmentation, Atlantic salmon, water temperature

## Abstract

**BACKGROUND:**

Cleaner fish play a crucial role in controlling caligid sea lice in Atlantic salmon aquaculture across the North Atlantic. However, their delousing performance varies due to multiple factors, including water temperature and cleaner fish size. Anecdotal reports of less‐pigmented sea lice have raised concerns that these variants may emerge as a response to cleaner fish selection pressure. Given the high adaptation potential of sea lice and their resistance to chemical treatments, these colour variants could challenge cleaner fish delousing strategies. A series of tank experiments cohabiting lice‐infected salmon with farmed cleaner fish investigated the effect of water temperature, hypopigmented ‘cryptic’ lice and the size of lumpfish on delousing. Delousing rates were calculated from changes in lice numbers over time.

**RESULTS:**

Ballan wrasse and lumpfish were both effective at delousing sea lice in Scottish summer and winter water temperatures with lice numbers significantly reduced after 4 days, showing that both species are effective delousers all year round. There was no significant difference in delousing between small (40 g) and large (80 g) lumpfish. Ballan wrasse were effective at delousing pigmented and cryptic lice but lumpfish were less effective at delousing cryptic lice, especially male lice.

**CONCLUSION:**

This study demonstrates the potential for year‐round efficacy of cleaner fish in salmon aquaculture, although it is highly dependent on the health and welfare of cleaner fish in commercial conditions. It also tested for the first time delousing of cryptic lice under experimental conditions, and results indicate that they should be considered in sea lice management strategies involving lumpfish. © 2026 The Author(s). *Pest Management Science* published by John Wiley & Sons Ltd on behalf of Society of Chemical Industry.

## Graphical abstract

Ballan wrasse and lumpfish effectively reduce sea lice on salmon year round, although lumpfish struggle with cryptic lice, highlighting new considerations for lice management.

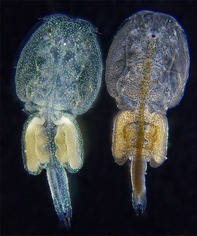

## 1 INTRODUCTION

Cleaner fish (ballan wrasse *Labrus begylta* and lumpfish *Cyclopterus lumpus*) are a key component of caligid sea lice management in Atlantic salmon (*Salmo salar*) aquaculture in North Atlantic countries. However, delousing performance in both species is variable because there are multiple factors affecting delousing that can be broadly categorised as environmental, genetic, husbandry and host/cleaner interactions.^1^, ^2^, ^3^, ^4^, ^5^, ^6^

Water temperature is widely recognised in the salmon aquaculture industry as one of the primary factors affecting health, welfare and delousing efficacy in cleaner fish, and typical deployment windows reflect the preferences of each species.^7^ Ballan wrasse are temperate fish, and a low metabolic rate and inactivity below 10 °C suggests that their delousing efficacy is limited in winter and at higher latitudes.^8^, ^9^ Below 6 °C they enter a state of torpor.^10^ By contrast, lumpfish continue to feed down to 4 °C,^11^ prefer temperatures below 10 °C,^12^ and are more susceptible to disease at higher temperatures.^13^

In terms of size, larger ballan wrasse (~75 g) have been observed to delouse more rapidly than smaller wrasse (~23 g).^14^ Although smaller lumpfish are thought to be more effective delousers than larger lumpfish (>140 g),^15^, ^16^ this has not been tested in controlled experimental conditions. At larger sizes (≥150 g), lumpfish in commercial salmon net pens can switch their diet to salmon pellets (industry observation). Given the rapid growth rates of lumpfish (1–2% day^−1^)^16^ this has implications for the effective delousing window for lumpfish.

The sea louse (*Lepeophtheirus salmonis*) has a high adaptation potential because of its high fecundity and short life cycle,^17^ wide abundance and high levels of gene flow between geographically distinct areas.^18^, ^19^, ^20^ Because of the historical widespread use of sea lice parasiticides in salmon aquaculture, genetic resistance to these chemicals has rapidly developed and dispersed across the entire North Atlantic,^21^, ^22^ especially in the absence of treatment rotation. Cleaner fish are now widely used as a component of integrated sea lice management strategies, and anecdotal reports suggest that less‐pigmented and unpigmented sea lice may emerge owing to cleaner fish selection pressure because cleaner fish are visual feeders and these variants may less visible. Hamre *et al*.^23^ found that lice pigmentation has a strong plastic (environmental) component, although an underlying adaptive (genetic) component is also possible. Imsland *et al*.^24^ found that delousing of Atlantic salmon in experimental pens did not affect the level of pigmentation in the lice population. Whether these hypopigmented lice are less visible to cleaner fish is unknown.

In this study, tank‐based trials were used to test the effects of water temperature and sea lice pigmentation on ballan wrasse and lumpfish delousing performance and to examine the influence of lumpfish size on lumpfish delousing.

## 2 MATERIALS AND METHODS

### 2.1 Experimental animals and environment

**Figure 1:**
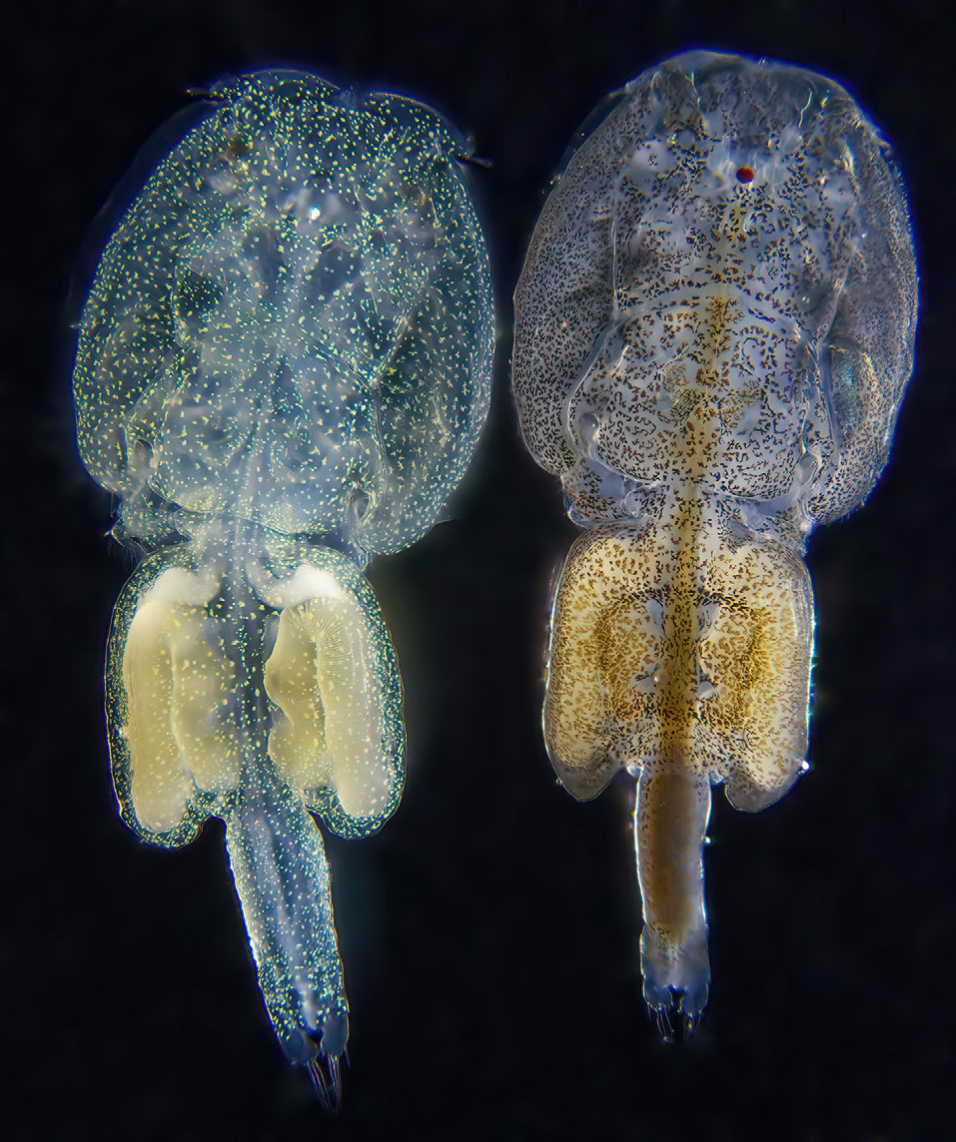
Adult female sea louse cryptic form (left) and pigmented form (right) cultured at the Marine Environmental Research Laboratory (MERL) showing the hypopigmented and normally pigmented cells of the two strains. Image by J Bron using Olympus TG‐6 with oblique illumination.

Trials were performed at the University of Stirling's Marine Environmental Research Laboratory (MERL), Machrihanish, in a flow‐through indoor tank system (12 × 750 L circular tanks) supplied with natural ambient seawater pumped ashore and a natural simulated photoperiod (17:7 h light/dark, dawn at 7 am). Sea lice were cultured on Atlantic salmon and originated from two strains maintained at MERL: normal pigmented lice (IoA‐02) sourced from Scotland and maintained at MERL since 2010 and hypopigmented ‘cryptic’ lice (IoA‐06) isolated as a Mendelian autosomal recessive trait from an in‐house pigmented strain originating from Scotland in 2014–2015. Although the exact mechanism of the deviation from normal pigmentation has not been investigated, observations suggest that it may be due to the development of xanthophores (yellow pigment chromatophores) rather than the normal melanophores (Fig. 1). Sea lice strains are cultured at MERL on Atlantic salmon with approximately ten generations per year.

Prior to the start of each trial, Atlantic salmon stocked in a 7‐m^3^ circular flow‐through tank at ambient temperature and salinity were subjected to a controlled sea louse copepodid infection challenge. Where salmon were infected with both pigmented and cryptic lice strains, a separate infection of each strain took place a week apart. The target infection level was ten lice per fish. Once the sea lice had developed to late chalimus stages (~6–8 weeks after infection, depending on water temperature), 30 salmon were randomly distributed into each experimental tank. Trials were initiated once the sea lice had developed to motile adult stages (1–2 weeks after stocking experimental tanks). Salmon were hand‐fed to visual satiation twice daily using a standard pelleted salmon feed (BioMar, Grangemouth, UK) with the ration set as a percentage of estimated tank biomass and varying according to fish size, water temperature and appetite, except during the trials when they were fed a minimal maintenance diet every 48–72 h to maintain good welfare. Farmed ballan wrasse and lumpfish were produced by Otter Ferry Seafish Ltd (Otter Ferry, UK) and transferred to MERL by road (~2 h transit). They were acclimated in 750‐L flow‐through tanks at ambient temperature and salinity containing fake kelp hides in the experimental system and were hand‐fed to visual satiation twice daily until required for delousing trials using BioMar Lumpfish Grower for lumpfish and BioMar Symbio for ballan wrasse. During the trials, they were fed a minimal maintenance diet every 48–72 h to maintain good welfare. Owing to the rapid growth rates of lumpfish and trials conducted at different times of year, a different cohort of lumpfish was used for each trial with varying size ranges because of the availability of fish.

### 2.2 Experimental design

All trials used a triplicate randomised tank design tested against a negative control (infected salmon without cleaner fish). Three cleaner fish were stocked into each treatment tank (10% cleaner‐to‐salmon ratio), which marked the start of each trial. No artificial shelters were provided in the tanks.

#### 2.2.1 Trial 1: Delousing in summer conditions and the effect of lumpfish size.

Three treatment groups were tested to compare ballan wrasse and lumpfish delousing of pigmented lice in summer conditions and two different sizes of lumpfish (ballan wrasse, 23.6 ± 3.9 g; small lumpfish, 40.8 ± 5.9 g; large lumpfish, 80.4 ± 11.0 g) against a negative control (total of 12 tanks, *n* = 3 per treatment). Atlantic salmon originating from Salmobreed eggs reared at Buckieburn smolt facility (Stirling, UK) had a mean weight of 867.2 ± 167.5 g with an infection level of 18.7 ± 6.2 adult lice per salmon across all tanks (5.5 ± 3.1 males, 13.2 ± 5.0 females). The trial period was 5 days, conducted from 12 to 16 August 2019, and water temperature and salinity during the trial were 14.5 ± 0.5 °C and 32.5 ± 0.5 ppt, respectively.

#### 2.2.2 Trial 2: Effect of sea lice pigmentation on summer delousing efficacy.

Three treatment groups were tested to compare ballan wrasse and lumpfish delousing of pigmented and cryptic lice (lumpfish 109.4 ± 25.3 g, wrasse 31.9 ± 8.6 g) against a negative control infected with both pigmented and cryptic lice (total of 12 tanks, *n* = 3 per treatment). A wrasse *versus* pigmented lice group was not included owing the limited number of tanks and because the efficacy of ballan wrasse against pigmented lice has already been assessed in previous experiments.^14^ Atlantic salmon originating from Salmobreed eggs and reared at Buckieburn smolt facility had a mean weight of 451.66 ± 62.18 g and infection levels of 9.5 ± 5.2 adult lice per salmon for pigmented lice (4.7 ± 2.8 males, 4.8 ± 3.4 females) and 4.4 ± 2.7 adult lice per salmon for cryptic lice (2.1 ± 1.5 males, 2.3 ± 1.9 females). The trial period was 8 days, conducted from 31 August to 7 September 2019, and water temperature and salinity during the trial were 13.0 ± 1 °C and 33 ppt, respectively. Based on previous studies,^14^ delousing was slower than anticipated in trial 1 (5 days), so trials 2 and 3 were extended to 8 days to capture more delousing activity.

#### 2.2.3 Trial 3: Delousing in winter conditions

Two treatment groups were tested to compare ballan wrasse and lumpfish delousing of pigmented lice in winter conditions (ballan wrasse, 30.7 ± 9.2 g; lumpfish, 63.5 ± 9.1 g) against a negative control (total of 9 tanks, *n* = 3 per treatment). Atlantic salmon originating from (MOWI Scotland, UK) eggs and reared at MOWI had a mean weight of 583.71 ± 124.37 g and an infection level of 8.5 ± 4.5 adult lice per salmon across all tanks (3.8 ± 2.3 males, 4.6 ± 3.1 females). The trial period was 8 days, conducted from 20 to 27 April 2020, and water temperature and salinity during the trial were 8.9 ± 0.1 °C and 33 ppt, respectively.

### 2.3 Sampling schedule

Sea lice counts were performed immediately prior to the introduction of cleaner fish and at 24‐h intervals for trial 1 and at 48 h (2 days), 96 h (4 days) and 168 h (7 days) for trials 2 and 3. For each sample, ten salmon were randomly sampled from each tank, sedated (MS‐222 at 100 ppm) and visually examined under a direct, bright light to record the number of adult male and female lice and their location on the fish. Salmon were allowed to fully recover in an aerated seawater bath before being returned to their original tank. At the last sampling point in each trial, salmon were also measured for body weight (± 0.1 g) and total length (± 1 mm) and assessed for eye, fin and skin damage. At the same time, all cleaner fish were culled, measured for body weight and total length and stomach contents dissected to count the number of lice present. There may be a risk that sea lice are dislodged and lost during repeat sampling of salmon, but the control groups in this study and Leclercq *et al*.^14^ suggests that any loss of lice is minimal and does not significantly affect the results.

All experimental procedures were approved by the University of Stirling Animal Welfare Ethical Review Board and were conducted under a Project Licence (PPL) issued by the UK Home Office under the Animals (Scientific Procedures) Act 1986.

### 2.4 Analysis

All data processing and analyses were conducted in R.^25^ Total adult male and female sea lice counts per salmon were averaged within each tank and at each time point. Data were checked for normality using the Anderson–Darling test and for homogeneity of variance using Levene's test and observation of residual plots. Differences in mean sea lice counts were assessed by repeated measures linear mixed‐effects model with replicate tanks as a random effect, and when a single time point was analysed, using linear mixed‐effects model with replicate tanks as a random effect using the nlme package. Tukey's post hoc tests were used for comparison between groups.

## 3 RESULTS

### 3.1 Effect of water temperature on delousing

**Figure 2:**
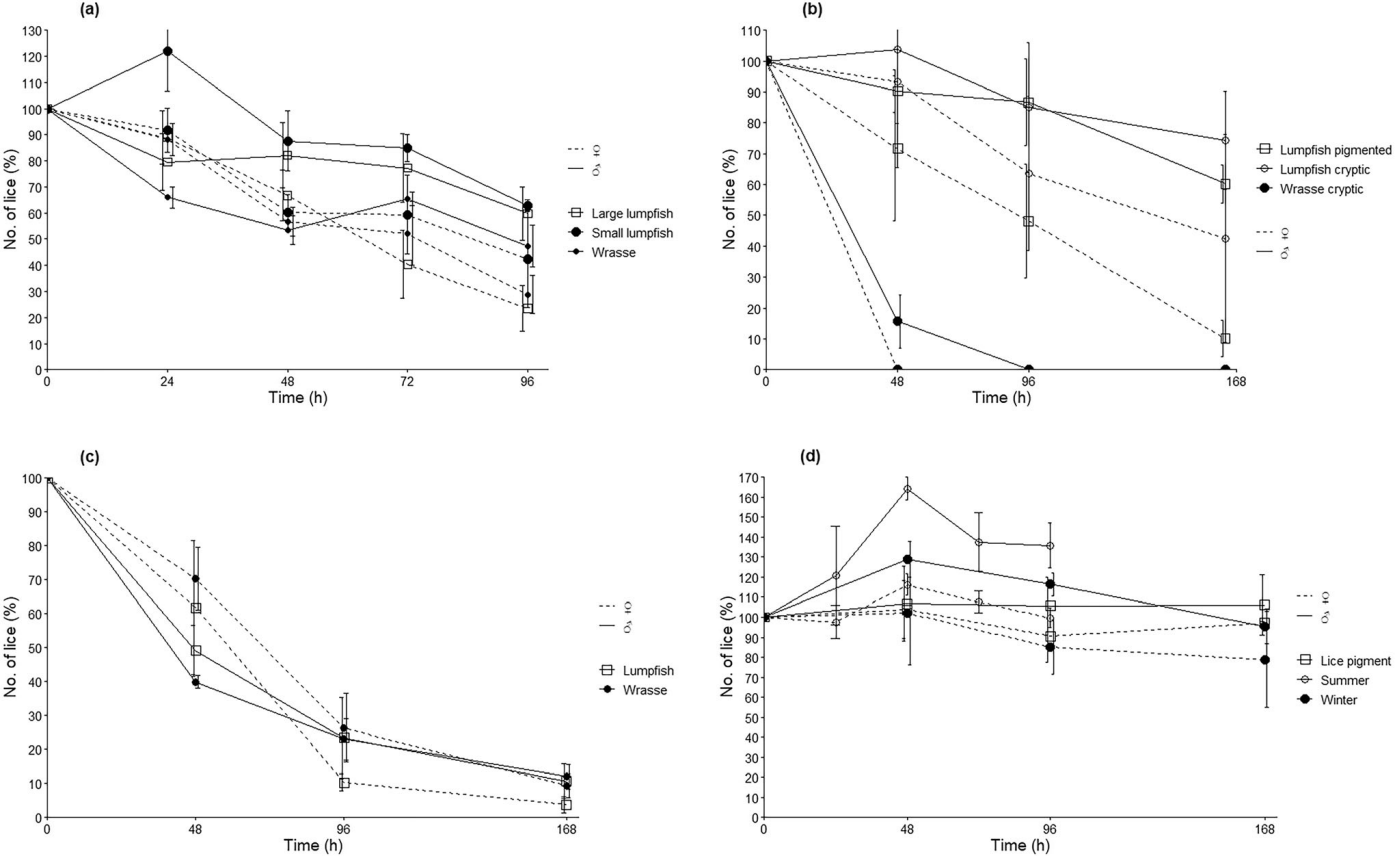
Change in mean lice numbers per treatment group over time relative to the control group at each time point in the summer trial (a), lice pigmentation trial (b) and winter trial (c), and control group lice numbers for all trials (d). Values are mean ± SE (*n* = 10 salmon per replicate per time point, *n* = 3 replicates per treatment), ♂ and ♀ refer to lice sex.

**Figure 3:**
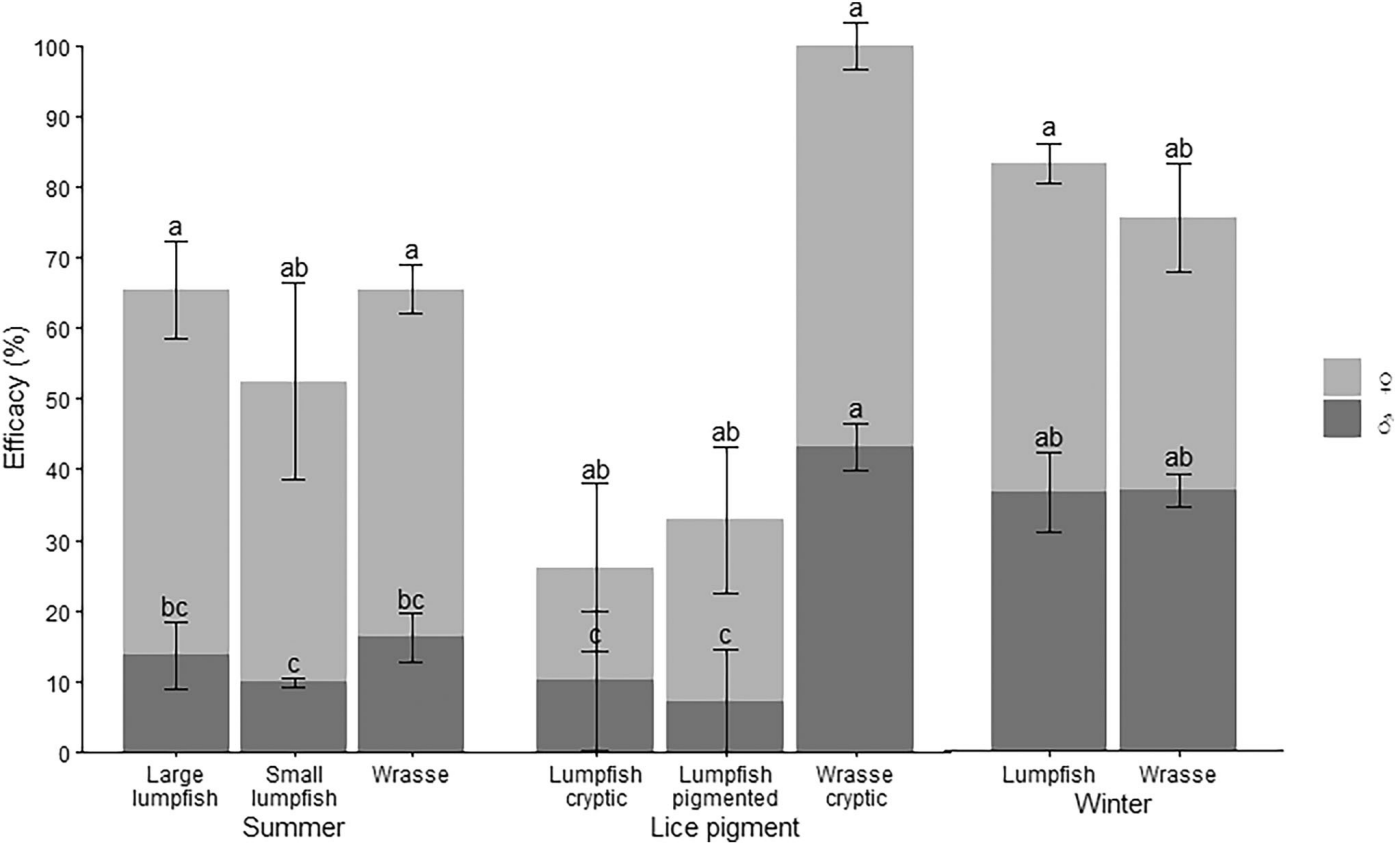
Delousing efficacy achieved within 96 h relative to the control group for the summer trial, lice pigmentation trial and winter trial. Values are mean ± SE (*n* = 10 salmon per replicate per time point, *n* = 3 replicate tanks per treatment), ♂ and ♀ refer to lice sex and superscript letters refer to statistical groups across all treatment groups for male and female lice calculated using a linear mixed‐effects model with tank replicate as a random effect and Tukey's post hoc test (*P* < 0.05).

In the summer trial (mean water temperature of 14.5 °C), all treatment groups were more effective at delousing female lice than male lice with numbers of female lice less than 50% after 96 h (*T*  (Test statistic)= −6.45, −6.10 and −8.40 for wrasse, small lumpfish and large lumpfish, respectively, df = 267, *P* < 0.001 compared to control) (Fig. 2(a)). Delousing rates for female lice over 96 h were 0.33 ± 0.05, 0.29 ± 0.17 and 0.37 ± 0.08 lice cleaner^−1^ h^−1^ for wrasse, small lumpfish and large lumpfish, respectively (Table 1). Male lice were deloused at a slower rate than female lice in all three treatment groups with delousing rates over 96 h of 0.07 ± 0.04, 0.03 ± 0.01, 0.05 ± 0.07 lice cleaner^−1^ h^−1^ for wrasse, small lumpfish and large lumpfish, respectively. The final delousing efficacy after 96 h in the summer trial was 62.3 ± 7.99% for large lumpfish, 51.2 ± 13.3% for small lumpfish and 64.08 ± 6.52% for wrasse (Fig. 3).

**Table 1:** Delousing rates (lice cleaner^−1^ h^−1^) over a 96‐h period for male, female and all lice in each treatment group

Trial	Group	Male lice	Female lice	All lice
Summer	Wrasse	0.22 ± 0.12^bcd^	0.98 ± 0.14^cd^	1.19 ± 0.22^e^
	Small lumpfish	0.08 ± 0.03^abc^	0.85 ± 0.50^bcd^	0.93 ± 0.52^e^
	Large lumpfish	0.16 ± 0.21^bcd^	1.10 ± 0.25^d^	1.26 ± 0.36^a^
Lice pigment	Wrasse *versus* cryptic	0.25 ± 0.06^bcd^	0.33 ± 0.05^ab^	0.58 ± 0.08^bcde^
	Lumpfish *versus* pigmented	0.10 ± 0.19^abcd^	0.39 ± 0.28^abc^	0.49 ± 0.21^abcd^
	Lumpfish *versus* cryptic	0.06 ± 0.11^abcd^	0.12 ± 0.14^a^	0.18 ± 0.25^abc^
Winter	Wrasse	0.33 ± 0.05^d^	0.39 ± 0.15^abc^	0.72 ± 0.17^abc^
	Lumpfish	0.31 ± 0.09^cd^	0.43 ± 0.03^abc^	0.74 ± 0.07^cde^

Values are given as mean ± SE. Superscript letters refer to statistical groups across all treatments for male and female lice calculated using linear mixed‐effects model with tank replicate as a random effect and Tukey's post hoc test (*P* < 0.05).

In the winter trial (mean water temperature 9.0 °C), lice numbers in all treatment groups were less than 30% after 96 h and less than 15% after 168 h (*T* = −5.33 and −5.74 for wrasse and lumpfish, respectively, df = 207, *P* < 0.001 compared with control) (Fig. 2(c)). Delousing rates after 96 h were similar for both species with rates for male lice of 0.11 ± 0.02 and 0.10 ± 0.03 lice cleaner^−1^ h^−1^ and rates for female lice of 0.13 ± 0.05 and 0.14 ± 0.01 lice cleaner^−1^ h^−1^ for wrasse and lumpfish, respectively (Table 1). Both species, but especially ballan wrasse, were observed following and delousing individual salmon, and some salmon had very low lice burdens (0–10% of their original lice burden with ballan wrasse) after 96 h, whereas other salmon retained up to 100% lice burden. The delousing efficacy after 96 h in the winter trial was 81.7 ± 8.0% for lumpfish, 75.0 ± 7.1% for wrasse (Fig. 3).

### 3.2 Effect of lumpfish size on delousing

Both male and female mean lice numbers were lower after 96 h with large lumpfish (80.4 ± 11.0 g) than with small lumpfish (40.8 ± 5.9 g) in the summer trial although there was no significant effect between groups (female lice remaining after 96 h was 23.3 ± 8.8% *versus* 42.1 ± 18.4% and male lice remaining after 96 h was 80.5 ± 14.0% *versus* 84.7 ± 2.9% for large and small lumpfish, respectively; *T* = −0.20 and 1.96 for male and female lice, respectively, df = 267, *P* > 0.05) (Fig. 2(a)).

### 3.3 Effect of cryptic lice on delousing

Ballan wrasse were able to delouse cryptic lice effectively with all female lice removed after 48 h and all male lice removed after 96 h (Figs 2(b) and 3), which equates to 48‐h delousing rates of 0.14 ± 0.04 and 0.22 ± 0.03 lice cleaner^−1^ h^−1^ for males and females, respectively. Lumpfish were less effective at delousing cryptic lice than pigmented lice (*T* = −7.02, df = 207, *P* < 0.001 for pigmented lice; *T* = −2.47, df = 207 and *P* < 0.05 for cryptic lice) with rates for male lice of 0.02 ± 0.04 and 0.03 ± 0.06 lice cleaner^−1^ h^−1^, and rates for female lice of 0.04 ± 0.05 and 0.13 ± 0.09 lice cleaner^−1^ h^−1^ for cryptic and pigmented lice, respectively (Table 1) (female lice 9.9 ± 5.7% *versus* 41.4 ± 32.8% and male lice 60.2 ± 6.2% *versus* 74.3 ± 16.0% remaining after 7 days for pigmented and cryptic lice, respectively) (Fig. 2(b)). The delousing efficacy after 96 h in the lice pigment trial was 25.7 ± 20.7% for lumpfish *versus* cryptic lice, 31.7 ± 5.8% for lumpfish *versus* pigmented lice and 100% for wrasse *versus* cryptic lice (Fig. 3).

**Figure 4:**
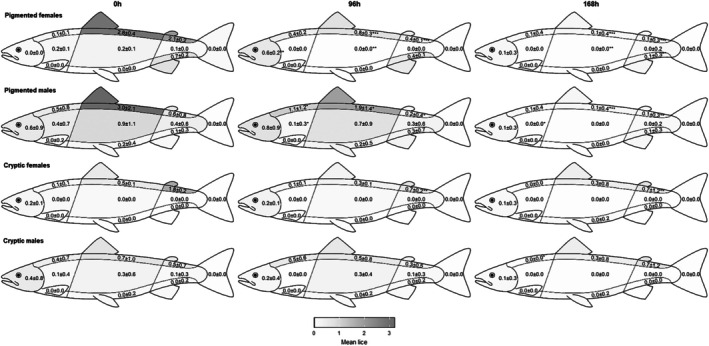
Lice pigmentation trial mean female and male lice numbers by location on salmon hosts at 0, 96 and 168 h for lumpfish that cohabited with salmon infected with pigmented or cryptic lice (wrasse data not shown as wrasse cleared all lice after 48 h). Values are mean ± SE (*n* = 10 salmon per replicate per time point, triplicate design), asterisks show significant differences in lice numbers from the 0 h sample (*P < 0.05, **P < 0.01, ***P < 0.001).

For lumpfish *versus* pigmented lice, lice numbers were significantly lower after 168 h compared with baseline numbers in the dorsal and ventral areas preferred by lice (behind dorsal, adipose and anal fins) (Fig. 4). However, for lumpfish *versus* cryptic lice, only female lice numbers were significantly lower after 168 h compared with baseline numbers in the dorsal area, but not the ventral area.

An increase in lice numbers over time was observed on the heads of the salmon (significant in pigmented female lice, *P* < 0.01) (Fig. 4) because of a forward migration of lice on the fish during the trial. The same forward migration of lice was seen with ballan wrasse.

### 3.4 Welfare assessment and gut contents

There was no evidence of physical damage in the salmon caused by cleaner fish, such as eye damage or fin nipping, and no such behaviours were observed in the cleaner fish. Some mild fin erosion, stomach abrasion and snout damage was seen in the salmon, but this is typical for salmon maintained in tanks and was present before the start of the trials. All cleaner fish were in good condition with no observed deformities or damage. No cleaner fish or salmon mortalities occurred during any of the trials.

In the summer trial, seven wrasse (78%), six small lumpfish (67%) and seven large lumpfish (78%) contained lice at the end of the trial. In the winter trial, seven lumpfish (78%) and no wrasse (0%) contained lice at the end of the trial. In the louse pigmentation trial, six lumpfish *versus* pigmented lice (67%), two lumpfish *versus* cryptic lice (22%) and no wrasse (0%) contained lice at the end of the trial.

## 4 DISCUSSION

Delousing performance in ballan wrasse and lumpfish is variable in commercial salmon net pens because of the influence of a wide range of factors. Although many of these factors, such as water temperature, currents and sea lice population dynamics, cannot be controlled, other factors, such as feeding, stocking densities and genetics continue to be refined to improve health, welfare and delousing. In this study, the effects of water temperature, lumpfish size and cryptic sea lice colour variants on delousing were assessed through a series of controlled tank experiments. Ballan wrasse and lumpfish were effective at delousing in summer and winter temperatures, although only ballan wrasse effectively deloused cryptic lice.

Both ballan wrasse and lumpfish were effective at delousing sea lice in both Scottish summer and winter water temperatures with lice numbers significantly reduced after 96 h (4 days) and a maximum delousing efficacy of 81.7% for lumpfish and 75.0% for ballan wrasse (winter trial). In comparison, Leclercq *et al*. reported that farmed ballan wrasse reduced sea lice burdens by 97.9 ± 0.1% after 84 h at 10.5 °C,^14^ although these fish were starved before and during experimentation. Wild ballan wrasse have a temperate distribution and wild lumpfish have a boreal distribution and their water temperature preferences reflect their geographical ranges.^7^ These results, however, show that both species can delouse effectively in a range of temperatures typical in Scottish waters. Delousing may be reduced at the lowest sea temperatures experienced in Scottish winters, especially in ballan wrasse because they are known to enter a state of torpor below 6 °C.^10^ However, lower activity below 10 °C^9^ did not affect delousing in the current study. This contrasts with previous studies suggesting that ballan wrasse efficacy may be limited at lower water temperatures below 10 °C due to decreased metabolic rate^9^ and showing that lumpfish prefer temperatures between 6 and 7 °C.^26^ A possible explanation for these differences is that although ballan wrasse and lumpfish activity, thermal preferences and welfare are correlated with water temperature, delousing performance is not and healthy cleaner fish are effective delousers at the range of temperatures tested in this study.

Although there were some compounding effects in the summer trial (large salmon size and high infection level), which may have affected the relative delousing rates, both lumpfish and wrasse were effective delousers in both Scottish summer and winter water temperatures. In the summer trial primarily (but apparent in all three trials), there was an increase in the number of lice on the control fish over time (Fig. 2(d)). It was noted that there were some pre‐adult lice at the start of the summer trial, and it is likely that this increase in counted lice through the trial is due to the development of these juvenile lice to adults. This means that delousing was higher than measured because of the continued recruitment of new lice to the adult population throughout the trials, although expressing delousing rates relative to the control group reduces this effect because all fish used in the trial were from the same lice infection.

A limitation of this study is the variation in salmon hosts sizes and lice burdens between trials with larger salmon and higher lice burdens in the summer trial than in the winter trial. Therefore, it is not surprising that the cleaner fish, especially the ballan wrasse (mean 23.6 g), did not fully delouse the salmon during the 96‐h trial. Nonetheless, all three groups of cleaner fish in the summer trial significantly reduced the number of lice on the salmon and had the trial continued, it is likely that they would continue to delouse and consume lice. In the winter trial with smaller salmon and lower lice burdens, ballan wrasse and lumpfish consumed most of the lice (>70%) within 96 h. These results provide an insight into the delousing abilities of cleaner fish related to host size and lice burden and show that both species are effective delousers even with large salmon hosts and high lice burdens.

Lumpfish are typically deployed in commercial salmon net pens when they are approximately 15–30 g^7^. Anecdotal industry reports suggest that smaller 30–40 g lumpfish are better delousers than larger lumpfish up to 100 g or more, and that delousing performance can quickly reduce because of the rapid growth rates of lumpfish. However, larger lumpfish are likely to be more robust than small lumpfish, which may also impact overall delousing performance. Lumpfish, being opportunistic feeders, tend to target other food sources including salmon pellets at larger sizes. In the current study, although there was no significant difference in delousing rates between small lumpfish (~40 g) and large lumpfish (~80 g), male and female lice numbers after 96 h cohabitation were both slightly lower with small lumpfish. Imsland *et al*.^15^, ^16^ suggested that delousing is reduced in lumpfish over 140 g but it could be that the 80 g fish tested are within the size range of effective delousers. When planning delousing experiments, it is essential to predict lumpfish growth rates to ensure the target weights to be tested coincide with the maturation of experimental lice, and further work should test larger lumpfish (>140 g) to determine the size that lumpfish become ineffective delousers.

In all three trials, female lice were deloused more than male lice, which is likely because of the larger size of female lice making them easier to spot by the visual‐feeding cleaner fish. Male lice are more pigmented than female lice^23^, ^24^ and can be very motile, rapidly moving across the surface of the fish, which could help them to evade cleaner fish. Although the egg strings of gravid females make them more attractive to cleaner fish,^14^ there were no gravid females present in the current study. Furthermore, the larger abdomen and genital segment of the female louse, which is not attached to the fish like the cephalothorax, may make them easier for cleaner fish to remove than the male louse with a much smaller abdomen and smaller attachment surface on the fish.

Although the presence of lice in cleaner fish stomachs was noted at the end of each trial, this is not an accurate method to estimate delousing efficacy because it does not account for lice that have already passed through the gut, especially in wrasse, which have no stomach and a short intestine. Indeed, no lice were found in the wrasse in the lice pigmentation and winter trials as the lice had already passed through the gut by the end of the trials. However, the presence or absence of lice in lumpfish gives an indication of the proportion of cleaner fish that engage in delousing behaviour and provides further evidence that lumpfish are ineffective against cryptic lice with only 22% of lumpfish containing cryptic lice compared with 67% of lumpfish *versus* pigmented lice.

Sea lice have a high evolutionary potential and rapidly adapt to strong selection pressures, such as from medicinal treatments.^21^, ^22^ The current study found that lumpfish are less effective at delousing a hypopigmented cryptic strain of lice, whereas ballan wrasse were equally effective at delousing pigmented and cryptic lice. The cryptic lice were isolated as a recessive trait from a laboratory‐cultured strain of pigmented lice. However, similar adaptive genetic mutations, as suggested by Hamre *et al*.,^23^ resulting in cryptic lice have been reported from several salmon farms in Scotland, which is not surprising if lumpfish are less effective against these lice and the lice are allowed to proliferate.

These results are in contrast to Imsland *et al*.^24^ who found that lumpfish delousing did not affect the pigmentation of sea lice and concluded that sea lice are not likely to adapt to reduce cleaner fish efficacy. However, the lice in Imsland *et al*.^24^ appear to be pigmented lice with varying levels of pigmentation due to plastic environmental adaptation and not hypopigmented cryptic lice resulting from a genetic mutation. Although the prevalence of mutations resulting in changes in levels of pigmentation in sea lice is unknown, it is possible that they are not uncommon, and where these mutations do occur, they could be allowed to proliferate through the selection pressure of lumpfish primarily targeting normally pigmented lice if other control strategies are not used. Albino lice (total lack of pigmentation) may also arise due to genetic mutations, but are likely to be extremely rare and unlikely to proliferate because of survival challenges related to albinism in wild populations.^27^

The position of the lice on the fish also has implications for delousing. Hamre *et al*.^23^ found that lice pigmentation varies according to light levels and lice on the dorsal surface of the fish, which are exposed to more light, are darker than lice on the ventral surface of the fish, which are exposed to less light. Although this helps normally pigmented lice to camouflage against the surface of the fish, this mechanism cannot occur in cryptic lice because of the reduced pigmentation. Lumpfish in the current study were less effective at delousing cryptic lice on the ventral surface and flank of the fish, although they did significantly reduce numbers of cryptic female lice on the dorsal surface. Although cryptic lice are well camouflaged against the lighter ventral surfaces and flanks of the fish, their lack of pigmentation may also camouflage the smaller male lice effectively against lumpfish on the darker dorsal surface of the fish.

In stark contrast to lumpfish, delousing in ballan wrasse was just as effective with cryptic lice and pigmented lice. The forward migration of lice towards the head of the salmon in both lumpfish and ballan wrasse could be a predation response as cleaner fish were often observed approaching salmon from behind, and this could influence the delousing dynamics and the contrast of lice against different areas of the salmon. This difference could be due to the different ecological niches and feeding behaviours of lumpfish and ballan wrasse. Lumpfish are omnivorous opportunistic feeders occupying a bentho‐pelagic niche.^28^ In comparison, ballan wrasse have a specialised feeding behaviour known as durophagy, meaning they consume hard‐shelled prey. They have extensible jaws and forward‐projecting teeth for gripping prey and molariform pharyngeal teeth for crushing prey.^29^ Although they mainly consume crustaceans and molluscs, algae and bryozoans also form part of their diet.^29^ They are also considered ‘nibblers’, eating small meals frequently rather than consuming large quantities at once, and this is reflected in their short digestive tract lacking a stomach and pyloric caeca.^30^ These characteristics make them well‐suited to consume sea lice from salmon, and although they are not obligate cleaners, such as some species of Labridae found on tropical reefs,^31^, ^32^ it is reasonable to conclude that they are facultative cleaners, potentially with innate cleaning behaviour. This may be why they are more effective at delousing cryptic lice than lumpfish.

The occurrence of hypopigmented cryptic lice at salmon farms has implications for the industry going forward. An increased prevalence of cryptic lice in farmed salmon may reduce the delousing efficacy of lumpfish. Although there are no documented reports of increased prevalence of cryptic lice where lumpfish are used, this is not surprising because cleaner fish performance is impacted by many factors, such as health, welfare and husbandry, and other treatments, such as medicinal or physical removal, are commonly used in conjunction with cleaner fish.^33^ Cleaner fish are one component of integrated sea lice management, and although other strategies, such as medicinal treatments and physical removal, may be effective irrespective of lice pigmentation, cryptic sea lice could proliferate where lumpfish are the only cleaner fish species used. Therefore, sea lice pigmentation should be monitored where only lumpfish are used and alternative treatments used when cryptic lice are detected. Alternatively, cohabiting lumpfish and ballan wrasse as routinely done by the industry is an effective defence against all colour variants of sea lice.

## 5 CONCLUSION

Cleaner fish are an effective biological treatment against sea lice, but their delousing efficacy is dependent on their health, welfare and delousing behaviour. Although this study explored aspects of delousing behaviour in a controlled tank environment, the results improve our understanding of temperature and fish size ranges for effective performance that can be used in salmon farms. However, although both ballan wrasse and lumpfish were effective at delousing lice in both summer and winter temperatures, their health and welfare can be adversely affected by seasonal changes in water temperatures and environmental conditions for each species, which will impact their delousing performance in commercial conditions.^7^, ^9^, ^13^ Therefore, a focus on the health and welfare of cleaner fish through effective husbandry underpinned by ongoing research in these areas should be a priority for responsible, ethical aquaculture practices and supporting economic sustainability. Although the reduced efficacy of lumpfish against cryptic lice demonstrates the risk of impaired performance against these phenotypes, the absence of sites with a high prevalence of cryptic lice suggests that current integrated pest management strategies do not allow them to proliferate. Nonetheless, this reduced efficacy raises questions regarding the contribution of lumpfish to sea lice management where cryptic lice occur. It would be prudent to increase surveillance of such phenotypes by recording them in routine lice counts. When properly cared for and used within the biological constraints of each species, however, ballan wrasse and lumpfish have the potential to contribute as a consistent and reliable component of integrated sea lice management in Atlantic salmon aquaculture.

## CONFLICT OF INTEREST

The authors declare no conflicts of interest.

## Data Availability

The data that support the findings of this study are available from the corresponding author upon reasonable request.
